# Foster children’s behavioral development and foster parent stress: testing a transactional model

**DOI:** 10.1007/s10826-017-0941-z

**Published:** 2017-11-15

**Authors:** Anouk Goemans, Mitch van Geel, Paul Vedder

**Affiliations:** Institute of Education and Child Studies, Leiden University, Leiden, The Netherlands

**Keywords:** Foster care, Foster children, Internalizing and externalizing behavior problems, Parental stress, Transactional model

## Abstract

The goal of this three-wave longitudinal study was to analyze foster parent stress and foster children’s internalizing and externalizing behaviors in a transactional framework. Participants in this study were 237 children in foster care in the Netherlands with, mostly, long placement durations (*M = *56.86 months, *SD* = 49.10 months). We examined concurrent, prospective unidirectional and bidirectional relations between foster children’s behavior and foster parent stress by using cross-lagged structural equation modeling and examined whether the results were stable across different subgroups of foster children. In contrast to our hypothesis, we found no bidirectional relations. There were unidirectional prospective pathways from foster children’s internalizing and externalizing problems to foster parent stress, but no significant prospective pathways from foster parent stress to foster children’s internalizing and externalizing problems. The results were fairly stable across different subgroups of foster children. The lack of bidirectional relations was unexpected given the presence of transactional relations in biological parent-child dyads. Foster parents seem not to influence their foster children when it comes to regulating problem behavior. Therefore, the question is whether foster parents can, in more general terms, help their foster children benefit from their improved home environment.

## Introduction

Foster care provides an opportunity to be raised in a family-type setting for children who can no longer be cared for by their parents. Yet, sometimes children’s development and adaptive functioning does not follow a positive course in foster care (Burns et al. 2004; Maaskant et al. 2014). Some foster children are characterized by problematic psychosocial functioning and their problems do not always diminish during their stay in foster care (see for an overview, Goemans et al. 2015). Furthermore, foster children’s problem behaviors, especially externalizing problems, are a major reason for placement breakdown (Oosterman et al. 2007). To improve the behavioral development of children in foster care and to reduce the risk of breakdown, it is important to longitudinally study conditions and processes that affect foster children’s behavioral development (Jackson et al. 2012). The interaction between parental stress and child development may be one of the relevant processes, because foster parent stress is likely to play a role in foster children’s behavioral functioning and foster placement breakdown (Brown and Bednar 2006; Farmer et al. 2005).

Foster parents take care of children who often have damaged attachment with their parents, initially caused by suboptimal parenting, neglect, or abuse (Greeson et al. 2011; Oswald et al. 2010). If the attachment to their parents was healthy, but residing with the parents was no longer possible, the bond with the biological parents may be broken due to placement in a foster family. The challenging consequences of this breach of attachment are not always alleviated by foster parents, even if they were trained and prepared for their task (Dorsey et al. 2008) or deliberately chose to be a foster parent (Rodger et al. 2006). The consequence is likely some kind of negativity in the interactions of the foster child in the foster family that corresponds to an extra burden on the foster parents, for instance in the form of an increase in the level of parental stress (Hurlburt et al. 2010; Jones and Morrissette 1999). This stress may negatively impact foster parents’ motivation to continue fostering and may lead to foster parent burnout (Brown and Bednar 2006; Farmer et al. 2005), which, in turn, might have a negative effect on foster children’s behavioral development.

Studies in the general population have repeatedly found that parental stress is related to children’s behavioral outcomes (Crnic and Low 2002; Deater-Deckard 1998): higher levels of stress corresponded to higher levels of behavioral problems. Studies on foster care have also shown that parental stress in foster parents is positively correlated with internalizing and externalizing behavior problems in foster children (Kelley et al. 2011; Murray et al. 2011). However, the correlational nature of these studies does not allow for conclusions about the directionality of the relations.

Longitudinal studies that focus exclusively on child-to-parent effects have shown that increased behavior problems are related to an increase of parent reported stress (Hurlburt et al. 2010; Vanderfaeillie et al. 2012). Although these longitudinal studies have improved our knowledge about the unidirectional effects from foster children to their foster parents, it is generally emphasized that the interactions between parenting and child development are bidirectional (Bell 1968; Bornstein 2009; Deater-Deckard 2004; O’Connor 2002) and need to be studied in a transactional framework (Neece et al. 2012; Sameroff 2009; Stone et al. 2016). The transactional model of child development (Sameroff 2009) is essential to an understanding of the dynamic, reciprocal processes by which children and their social and material environments influence each other throughout development. The transactional model concentrates both on single factors and on the dynamic interplay between factors. Core to the transactional model is the analytic emphasis placed on the bidirectional, interdependent effects of the child and the environment (Bornstein 2009; Sameroff 2009). No study to date has tested the mutual associations with respect to parental stress and children’s behavioral development in foster care. Moreover, studies examining these associations and underlying processes by three-term interactions (i.e., child-parent-child or parent-child-parent) are lacking.

**Fig. 1:**
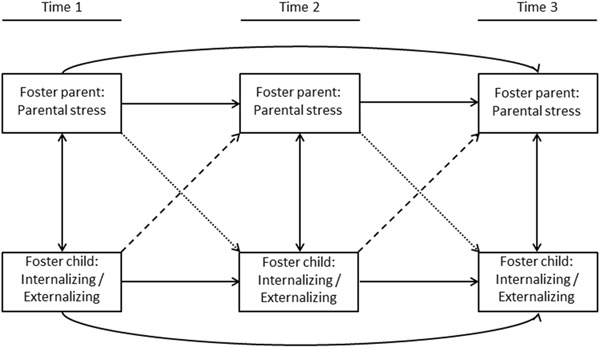
Structural equation model for testing foster children’s behavioral functioning and foster parent stress in a transactional perspective

The aim of the current study is therefore to examine in a three-wave longitudinal study the behavioral development of foster children from a so-called transactional perspective (Sameroff 2009). We will examine the interplay between foster children’s internalizing and externalizing behaviors and foster parent stress by using cross-lagged structural equation modeling. We will test and compare three models (depicted in Fig. 1) on the concurrent, unidirectional and bidirectional relations between foster children’s behavioral functioning and foster parent stress. In line with the results from studies on the general population (Neece et al. 2012; Stone et al. 2016), we hypothesize concurrent, prospective unidirectional, and bidirectional positive relations between foster children’s internalizing and externalizing behavior problems and foster parent stress. In addition, we hypothesize that the bidirectional model will fit the data better than a model with only concurrent relations or a model with either only parent-to-child or only child-to-parent relations. As a last step, we will examine whether the results are stable across different subgroups of foster children, because it has been previously shown that age (Oosterman et al. 2007), placement history (Oosterman et al. 2007) and duration of the placement (Goemans et al. 2015) are important variables with respect to foster children’s functioning. We will perform multigroup analyses on these variables to study the stability of our results across different groups of foster children.

## Method

### Participants

Participants in this three-wave longitudinal study were 237 foster parents of foster children residing in regular foster care in the Netherlands. The foster children were between 4 and 17 years old (*M* = 10.69, *SD* = 3.71) at the first measurement, and included 105 girls (44.3%) and 132 boys (55.7%). At the first wave, foster children had experienced an average of 1.18 previous foster placements (*SD* = 1.37), and the mean time in the current foster placement was 62 months (*SD* = 49.36 months). The majority of foster children resided in mandated foster care (80.6%) and with non-kinship foster families (67.1%). In over half of the foster families, there were no other foster children present (55.3%). Foster families were mostly two-parent families (92.0%). Almost one fifth of foster mothers had completed primary or junior high school (18.1%), but most had completed senior high school (36.7%) or a university of applied sciences (33.8%). Less than 10% (8.9%) graduated from an academic university. The educational backgrounds of foster fathers resembled those of mothers, with 11.4% having completed primary or junior high school, 37.6% senior vocational high school, and 30% a university of applied sciences. In addition, 13.9% of foster fathers held an academic university degree. Similar percentages regarding education can be found in the general population (Statistics Netherlands 2013). Approximately 59.5% of foster mothers and 81% of foster fathers had a job. Foster mothers worked fewer hours a week than foster fathers.

The initial sample that participated in Wave I consisted of 549 foster families. Excluded from the study were foster children who fell outside of the age range of 4–17 years (*N* = 30), for whom we had no behavioral functioning scores (*N* = 43), or who resided in part-time foster care (*N = *45). We compared our final sample with the total population of Dutch foster families and foster children to examine the representativeness of our sample. This was done based on the figures reported in the yearly factsheet of all Dutch foster care institutions (Pleegzorg Nederland 2015). Our sample had a smaller proportion of young children (5–11 years) than the total population of Dutch foster families, but other age groups were representative. There were slightly more boys in our sample (55.7%) compared to an almost equal (49 out of 51) boys/girls distribution in the total population of foster children. With respect to the duration of the current placement, our sample had a higher proportion of longer lasting placements (>2 years; 70%) than the total population (47%). The large number of stable placements in our sample might be a consequence of our invitation that emphasized the longitudinal nature of the study. Foster families who took care of a foster child with the expectation of a short-term stay, knowing that the study would last a year while their foster child might leave from care within the year, may have decided to refrain from participation. Regarding foster family characteristics, we included fewer kinship foster families (32.1% vs. 41.0%) and more mandated placements (80.6% vs. 72.0%).

The final sample consisted of 431 foster families. Of these families, 212 foster families also participated in Wave II (attrition rate 50.8%) and 180 in Wave III (attrition rate 58.2%). Reasons for attrition were mostly unknown (*N = *212), but reunification with birth parents (*N* = 17), placement change to another foster family (*N* = 9), placement change to residential or group care (*N* = 8), and leaving foster care because of independent living of the foster child (*N = *5), were communicated as reasons for dropout. Little’s (1988) Missing Completely at Random (MCAR) test indicated that the missing data were missing completely at random (*χ* ^2^ (47) = 56.542, *p* = 0.16). The final sample of foster children (*N = *237) was compared to the foster children that both dropped out after Wave I and did not participate in Wave III (*N = *194) on demographic variables (age, gender, kinship/non-kinship, placement duration, placement history, legal framework) and variables substantively relevant to the study (internalizing, externalizing and prosocial behavior, parenting, parental stress). T-tests and chi-square tests revealed only one difference between the two groups: there were fewer voluntary placements in the sample of remaining foster children (*χ* ^2^ (1) = 8.241, *p* < 0.00). Later in this text (see Analyses) we explain how we achieved a final sample size of 237 and that we used Full Information Maximum Likelihood (FIML) estimation to handle missing data.

### Procedure

The study was approved by the Leiden University Ethics Review Board. All foster care agencies in the Netherlands (*N = *28) were asked to participate in a three-wave longitudinal study on the development of children in foster care. Seven agencies (25.0%) agreed to participate. The main reason for foster care agencies to not participate was that they already participated in other foster care related research projects and wanted to prevent a research overload for their foster families. For foster care agencies that agreed to participate, foster parents were informed about the goal of the study and consent was requested by the foster care agencies. The researchers received the contact information for consenting foster parents. In most cases, researchers sent email invitations to request participation. In case of foster families without access to the Internet or when agencies did not know e-mail addresses, foster families received their invitation by regular mail (5.2%).

Children were followed for 12 months throughout their stay with their foster parents. During these 12 months, we established one baseline measurement (Wave I: October 2014), and two subsequent measurements separated by 6-month intervals (Wave II: April 2015, Wave III: October 2015). For the first wave, 1387 foster families were invited by the authors to participate in the study and to complete a questionnaire about the foster placement. For each of the three waves, foster parents were asked to complete an online questionnaire containing questions about the foster child, the foster family and the foster placement. Each wave, two reminders to fill out the questionnaire were sent, at a two-week interval. The online questionnaire was closed three weeks after the last reminder. All foster parents who participated in Wave I (*N* = 549, response rate 39.6%) were invited to participate in both Wave II and Wave III. For Wave III they were invited even if they had not participated in Wave II. The same foster parent completed the measures at every time point. Both foster parents could be primary caregivers, so either of the two foster parents could complete the questionnaire. Research has shown that there is strong agreement in reporting between foster mothers and foster fathers (McAuley and Trew 2000; Stanger and Lewis 1993).

### Measures

#### Socio-demographics and foster care characteristics

Foster parents provided information about the foster child (e.g., age, gender, placement history, duration of the current placement), foster family (e.g., kinship or non-kinship, single or two-parent household) and foster placement (e.g., legal framework, parental visitation).

#### Behavioral functioning

To measure the behavioral development of the foster children, the Dutch version (Van Widenfelt et al. 2003) of the Strengths and Difficulties Questionnaire (SDQ) for parents was used (Goodman 1997). The SDQ consists of 25 items that can be answered on a 3-point Likert scale ranging from 0 (not true) to 2 (very true). As previously suggested (Goodman et al. 2010), the 25 items were combined into three subscales: internalizing behavior problems, externalizing behavior problems, and prosocial behavior. Only the internalizing and externalizing subscale are used in this study. The internalizing behavior problems subscale consists of ten items covering emotional and peer problems. Example items are: “has many worries or often seems worried” and “picked on or bullied by other children”. The externalizing behavior problems subscale is formed by combining the ten items for conduct and hyperactivity problems. Example items are “often lies or cheats” and “restless, overactive, cannot stay still for long”. The SDQ is a well-validated screening instrument (Achenbach et al. 2008; Van Widenfelt et al. 2003) and the subscales have been shown to have good convergent and discriminant validity (Goodman et al. 2010). Studies using the Dutch version of the SDQ found acceptable to good psychometric properties (Muris et al. 2003; Van Widenfelt et al. 2003). In the current study, Cronbach’s alphas were 0.77, 0.79, and 0.76 for internalizing and 0.85, 0.82, and 0.84 for externalizing problems for the three waves respectively.

#### Parenting stress

Parenting stress was measured with the abbreviated version of the Nijmeegse Ouderlijke Stress Index verkort (NOSI-K; De Brock et al. 1992), which is based on the Parenting Stress Index (PSI; Abidin 1990) and has been previously used in research with foster parents (Maaskant et al. 2016; Timmer et al. 2006). The NOSI-K consists of 25 items rated on a 6-point Likert scale ranging from 1 (totally disagree) to 6 (totally agree). A sample item is: “Child does things that bother me a great deal”. Furthermore, the NOSI-K has been found to have high internal consistency (De Brock et al. 1992; Haskett et al. 2006). Cronbach’s alphas in the current study were 0.95, 0.96, and 0.96 for the three waves, respectively.

### Data Analyses

Statistical modeling of transactional processes requires monitoring of factors in the child and context over time, in order to provide a basis for determining when these factors affect and are changed by each other. Our 1 year, three-wave longitudinal design allows to study two-term unidirectional relations (i.e., child affects parents or parent affects child), as well as three-term reciprocal relations (i.e., the child changes the parent and is in turn changed by the changed parent, or the parent changes the child and in turn is changed by the changed child) (Bornstein 2009). Structural equation modeling using EQS 6.2 (Bentler 2004) was used to test a parallel set of three structural equation models (see Fig. 1). Baseline models (referred to as model 1) were tested to investigate the concurrent relations between parental stress and children’s behavioral functioning. Stability effects (i.e., regression lines between the same constructs over time) were included for child behavior problems over time as well as for parental stress over time. In the second model, either cross paths from parent-to-child (model 2a; Fig. 1: dotted lines) or child-to-parent (model 2b; Fig. 1: dashed lines) were added to test the longitudinal one-way effects of parental stress to foster children’s behavioral functioning, or vice versa. In the final model (model 3), both parent-to-child and child-to-parent cross paths were added to test the bidirectional relations between parental stress and foster children’s internalizing and externalizing behavior problems.

Multigroup analyses were performed for age (4–10 years vs. 11–17 years), placement history (no previous placements vs. one or more previous placements) and duration of the placement ( < 4 years vs. ≥ 4 years) (Goemans et al. 2015; Oosterman et al. 2007). We tested which model fit best, based on the fit indices, for both groups simultaneously. Subsequently, we analyzed whether regression loadings were similar for the different subgroups by constraining the regression loadings to be equal. If this resulted in a decrease in model fit, this indicated that the bidirectional regression loadings differed between the subgroups of foster children. If the model fit remained equally good, this indicated that the bidirectional regression loadings were similar.

Goodness-of-fit statistics are reported for each individual model in Table 2. First, Yuan–Bentler Chi-square value (Y-B*χ* ^2^), a rescaled chi-square statistic for non-normal missing data (Yuan and Bentler 2000), is reported and should be non-significant. Second, the Comparative Fit Index (CFI) represents whether the hypothesized model fits the data better than a more restricted baseline model. CFI values > .95 are considered as good (Hu and Bentler 1999). Third, the Root Mean Square Error of Approximation (RMSEA) reflects whether the a-priori model successfully reproduces data patterns. RMSEA values less than .08 indicate acceptable model fit and values below .05 indicate good fit (Hu and Bentler 1999). Fourth, the Akaike Information Criterion (AIC) concerns the issue of parsimony in the assessment of model fit and can be used in comparison of two or more models with smaller values representing a better fit of the hypothesized model (Byrne 2008). To examine whether the goodness-of-fit statistics improved for the consecutive models, the Chi-square difference test, corrected for the use of Yuan-Bentler Chi-square (Byrne 2008; Satorra and Bentler 2001) was performed. A significant Chi-square difference test means that the consecutive nested model explains the data better than the previous, more parsimonious, model. In case of a non-significant chi-square difference test, the more parsimonious model is preferred (Bentler and Mooijaart 1989). In addition, the differences in CFI and RMSEA between the models are reported. If the CFI increases more than .01, this means that there is substantial increase in model fit.

As in many longitudinal studies on foster children (Jackson et al. 2012), attrition was a problem in the current study. Internalizing and externalizing problems both had 13.9 and 24.1% missing in the second and third waves respectively. For parental stress, this was 0.4%, 16.5%, and 24.9% missing for the three consecutive waves. In line with previous studies (Keijsers et al. 2012; Sulik et al. 2015) and as recommended by researchers (Enders and Bandalos 2001; Jeličić et al. 2009), the current study used FIML estimation to not only include foster children who participated in each of the three waves (*N* = 156), but also foster children who participated in both Wave I and Wave II, but not in Wave III (*N = *56), or in both Wave I and Wave III, but not in Wave II (*N* = 25). This resulted in a final sample of 237 foster children. Within FIML, missing data are not replaced or imputed, but handled within the analysis model by the FIML method. The transactional model in our study is estimated by a FIML method, using all of the available data to identify the parameter values that have the highest probability of producing the sample data (Baraldi and Enders 2010; Byrne 2008; Enders 2001; Graham 2009). It has been suggested that FIML is an appropriate method to handle missing data (Graham 2009). In addition, FIML is available in all major SEM packages, including EQS. All statistics reported in this article were estimated using robust FIML.

## Results

Descriptive statistics and correlations for all study variables are presented in Table 1. The mean total behavior problems (internalizing and externalizing behaviors) of our sample (*M*_*WaveI*_ = 12.63, *SD*_*WaveI*_ = 7.06; *M*_*WaveII*_ = 12.83, *SD*_*WaveII*_ = 6.99; *M*_*WaveIII*_ = 12.17, *SD*_*WaveIII*_ = 6.89) fell within the borderline range following the Dutch norm cut-off scores (Goedhart et al. 2003). Of all foster children, 43.0%, 41.7%, and 46.7% fell within the normal range (range: 0–10) for the three consecutive waves, whereas 14.0%, 14.2%, and 16.6% fell within the borderline range (range: 11–13), and 43.0, 44.1, and 36.7% within the clinical range (range: 14–40) with regard to their SDQ total behavior problems score. Based on the non-clinical Dutch norm cut-off scores of the NOSI-K for mothers (De Brock et al. 1992), the mean total parental stress scores fell within the average range (range: 43–61). Parental stress was below average (range: 0–42) for 38.1%, 42.4%, and 39.3% of the parents for the three waves respectively, whereas 22.9%, 15.9%, and 18.0% of the foster parents scored ‘average’ levels of parental stress, and 39.0%, 41.9% and 42.7% scored above average (range: 62–150) for the three consecutive waves respectively. Cross-time correlations were 0.76 and 0.77 for internalizing, 0.85 and 0.79 for externalizing behavior, and 0.76 and 0.76 for parenting stress.

**Table 1:** Pearson correlations between the SDQ and NOSIK for each wave (T1, T2, T3)

	*M* (*SD*)	1	2	3	4	5	6	7	8	9
1. T1 SDQ internalizing	5.03 (3.83)									
2. T1 SDQ externalizing	7.60 (4.64)	0.387**								
3. T1 NOSIK	56.38 (24.46)	0.468**	0.583**							
4. T2 SDQ internalizing	5.13 (4.00)	0.771**	0.323**	0.384**						
5. T2 SDQ externalizing	7.52 (4.44)	0.339**	0.847**	0.521**	0.368**					
6. T2 NOSIK	56.84 (26.12)	0.338**	0.533**	0.780**	0.419**	0.597**				
7. T3 SDQ internalizing	5.02 (3.80)	0.764**	0.283**	0.403**	0.811**	0.293**	0.346**			
8. T3 SDQ externalizing	7.14 (4.40)	0.353**	0.784**	0.453**	0.374**	0.850**	0.512**	0.410**		
9. T3 NOSIK	57.46 (26.44)	0.411**	0.505**	0.773**	0.471**	0.542**	0.797**	0.465**	0.590**	–

*p < .05; **p < .01

### Parental Stress and Internalizing Problems

**Fig. 2:**
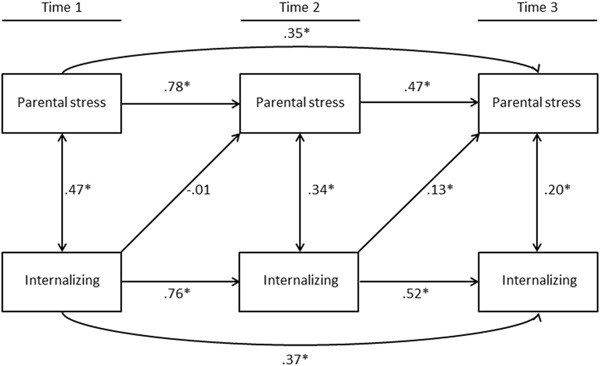
Structural equation model (2b-model) with cross paths between children’s internalizing behavior problems and foster parent stress *p < 0.05

The baseline model (model 1) to investigate the concurrent relations between parental stress and children’s internalizing behaviors was examined, and fit the data well, Y-B*χ*2 (*df* = 6) = 8.005, *p* = 0.238, CFI = 1.000, RMSEA = 0.000, 90% CI (0.000, 0.081). The consecutive models, wherein parent-child effects (model 2a), child-parent effects (model 2b), or the bidirectional relations (model 3) were tested, also fit the data well. The goodness-of-fit statistics are reported in Table 2. Despite overall good model fit, significant cross-lagged effects were only present for children’s internalizing problems at Wave II to parental stress at Wave III (*β* = .13, *p* < 0.05), and not for parental stress to children’s internalizing problems. Standardized coefficients for each model are reported in Table 3. In order to test whether the bidirectional model fit the data better than the model with only concurrent relations, or the models with either parent-to-child or child-to-parent effects, difference tests were performed for the consecutive models. The results are reported in Table 2. It appeared that the model with cross-lagged paths from child-to-parent (model 2b) fit better than the baseline model (ΔY-B*χ*2 (*df = *2) = 7.308, *p* = 0.026). The bidirectional model (model 3) did not fit better than model 2b (ΔY-B*χ*2 (*df = *2) = 1.288, *p* = 0.525). For reasons of parsimony (Bentler and Mooijaart 1989), model 2b is preferred over the bidirectional model. This model is depicted in Fig. 2.

**Table 2:** Structural equation models on the concurrent (model 1), parent-to-child (model 2a), child-to-parent (model 2b) and transactional relations (model 3) between parental stress and internalizing and externalizing behavior problems

							Difference tests^a^				
Model	Y-B*χ*2	*df*	*p*	CFI	RMSEA (90% CI)	AIC	ΔCFI	ΔRMSEA	ΔY-B*χ*2	Δ*df*	*p*
*Internalizing behavior problems*											
Model 1: concurrent	8.005	6	0.238	1.000	0.000 (0.000, 0.081)	−4.00					
Model 2a: parent-to-child	6.635	4	0.156	1.000	0.019 (0.000, 0.102)	−1.37	0.000	0.019	1.260	2	0.533
Model 2b: child-to-parent	1.973	4	0.741	1.000	0.000 (0.000, 0.060)	−6.03	0.000	0.000	7.308	2	0.026
Model 3: transactional	0.874	2	0.648	1.000	0.000 (0.000, 0.086)	−3.13	0.000	0.000	1.288	2	0.525
*Externalizing behavior problems*											
Model 1: concurrent	11.986	6	0.068	0.999	0.047 (0.000, 0.105)	−0.01					
Model 2a: parent-to-child	9.326	4	0.053	0.999	0.058 (0.000, 0.125)	1.33	0.000	0.011	2.614	2	0.217
Model 2b: child-to-parent	2.398	4	0.663	1.000	0.000 (0.000, 0.061)	−5.60	0.001	0.047	11.295	2	0.005
Model 3: transactional	1.657	2	0.437	1.000	0.000 (0.000, 0.107)	−2.34	0.002	0.000	0.972	2	0.615

Y-B*χ*2 Yuan-Bentler chi-square, *df* degrees of freedom, *CFI* comparative fit index, *RMSEA* root mean square error of approximation, 90% CI 90% confidence interval, *AIC* akaike information criterion

^a^ Difference tests are performed for the consecutive models. The 2a/2b-model are compared with the 1-model. The 3-model is compared with the best fitting 2-model

**Table 3:** Standardized coefficients for the structural equation models on the concurrent (model 1), parent-to-child (model 2a), child-to-parent (model 2b) and transactional relations (model 3) between parental stress and internalizing (int) and externalizing (ext) behavior problems

	Internalizing behavior problems				Externalizing behavior problems			
	Model 1	Model 2a	Model 2b	Model 3	Model 1	Model 2a	Model 2b	Model 3
*Stability effects*								
T1 parental stress → T2 parental stress	0.77*	0.78*	0.78*	0.78*	0.77*	0.78*	0.71*	0.72*
T2 parental stress → T3 parental stress	0.50*	0.51*	0.47*	0.47*	0.47*	0.49*	0.42*	0.44*
T1 parental stress → T3 parental stress	0.38*	0.37*	0.36*	0.35*	0.40*	0.39*	0.39*	0.38*
T1 int/ext → T2 int/ext	0.77*	0.75*	0.76*	0.74*	0.84*	0.82*	0.85*	0.84*
T2 int/ext → T3 int/ext	0.51*	0.49*	0.52*	0.51*	0.63*	0.60*	0.66*	0.64*
T1 int/ext → T3 int/ext	0.38*	0.37*	0.37*	0.37*	0.25*	0.24*	0.24*	0.23*
*Concurrent effects*								
T1 parental stress ↔ T1 int/ext	0.47*	0.47*	0.47*	0.47*	0.59*	0.59*	0.59*	0.59*
T2 parental stress ↔ T2 int/ext	0.34*	0.34*	0.34*	0.33*	0.40*	0.40*	0.40*	0.40*
T3 parental stress ↔ T3 int/ext	0.22*	0.22*	0.20*	0.20*	0.40*	0.41*	0.40*	0.39*
*Cross-lagged effects*								
T1 parental stress → T2 int/ext		0.04		0.05		0.04		0.02
T2 parental stress → T3 int/ext		0.03		0.02		0.07		0.04
T1 int/ext → T2 parental stress			−0.01	−0.01			0.11*	0.10
T2 int/ext → T3 parental stress			0.13*	0.12*			0.12*	0.11*

*p < 0.05

To test whether these results were stable across different subgroups of foster children, we performed several multigroup analyses. As was true for the entire group, parent-to-child effects were absent for the different subgroups, and model 2b fit best for each of the subgroups. Unidirectional regression loadings from children to parents were similar for foster children with different placement histories (no previous placements vs. one or more previous placement) and for foster children with different lengths of stay in the foster family ( < 4 years vs. > 4 years). Unidirectional regression loadings from children to parents appeared to be different for younger (4–10 years) and older (11–17 years) foster children. For older children, similar to the entire group of foster children, children’s internalizing problems at Wave I were not significantly related to foster parent stress at Wave II. For younger children, there was a significant child-to-parent effect from Wave I to Wave II (*β* = 0.18, *p* < 0.05).

### Parental Stress and Externalizing Problems

**Fig. 3:**
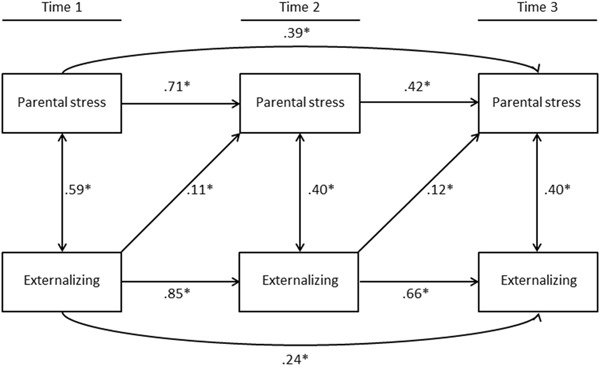
Structural equation model (2b-model) with cross paths between children’s externalizing behavior problems and foster parent stress *p < 0.05

As was true for the baseline model for internalizing problems, the baseline model on the concurrent relations between parental stress and externalizing behavior problems fit the data well (Y-B*χ*2 (*df* = 6) = 11.986, *p* = .068, CFI = 0.999, RMSEA = 0.047, 90% CI (0.000, 0.105)). The consecutive model, wherein the cross-lagged paths between either parent-child (model 2a) or child-parent (model 2b), and the transactional model, with cross-lagged paths between both parent-child and child-parent, also fit the data well. Table 2 shows the goodness-of-fit statistics for each model. Significant cross-lagged effects were again only present for children’s externalizing problems to parental stress from Wave I to Wave II (*β* = 0.11, *p* < 0.05) and from Wave II to Wave III (*β* = 0.12, *p* < 0.05), but not for parental stress to children’s externalizing problems. Table 3 gives an overview of the standardized coefficients of each model. In line with the model on internalizing behaviors, chi-square difference tests indicated that the model with cross-lagged paths from child-to-parent (model 2b) fit the data better than the baseline model (ΔY–B*χ*2 (*df = *2) = 11.295, *p* = 0.005), and better than the third model in terms of parsimony. Model 2b is depicted in Fig. 3.

Multigroup analyses were performed to test the stability of the findings. In line with the results of the entire group of foster children, for both younger and older children and for children with different lengths of stay in the foster family, there were no parent-to-child effects and model 2b fit best. Furthermore, unidirectional regression loadings from children to parents were similar for these subgroups of foster children. However, for foster children who differed in their experience of placement history, the multigroup analyses showed two differences. First, the bidirectional model (model 3) fit better for the subgroup of foster children who had experienced one or more placements. Although there was no significant parent-to-child effect from Wave I to Wave II, there was a significant parent-to-child effect from Wave II to Wave III (*β* = 0.20 *p* < 0.05) for foster children with a placement history. Second, unidirectional regression loadings from children to parents for model 2b differed for foster children with different placement histories. In contrast to the findings of the entire group, child-to-parent effect from Wave I to Wave II were not present for the group who did not experience previous placements. Similar to the results of the entire group, child-to-parent effects from Wave II to Wave III were present for both groups.

## Discussion

The primary goal of this study was to test relations between foster parent stress and foster children’s internalizing and externalizing behavior in a transactional framework by using cross-lagged structural equation modeling (Farmer et al. 2005; Goemans et al. 2016; Jones and Morrissette 1999; Sameroff 2009). The results of this study suggest that foster children influence their foster parents, but that foster parents do not influence their foster children. Both foster children’s internalizing and externalizing behavior problems are significantly related to foster parent stress, with higher levels of behavior problems related to higher levels of parental stress. In other words, there is a unidirectional prospective relationship from foster children’s internalizing and externalizing problems to foster parent stress.

There were no significant prospective pathways from foster parent stress to foster children’s behavioral functioning. This does not mean that foster parents did not experience parental stress. In comparison to a sample of Dutch parents from the general population (Goemans et al. 2016), the foster parents in the current study reported higher stress levels. Although approximately 40% of the foster parents in the current study reported low levels of parental stress, the same percentage of foster parents in our sample experienced above-average parental stress levels. Nevertheless, foster parent stress apparently does not exert an influence on foster children’s behavioral functioning. This result is in contrast with our hypothesis, which was based on previously established transactional relations in the general population, wherein prospective pathways from parental stress to children’s behavioral functioning have been reported (Neece et al. 2012; Stone et al. 2016). Transactional processes characteristic of children and their biological parents are apparently not characteristic of foster children and their foster parents with respect to parental stress and children’s behavioral functioning. A lack of transactional relations in foster care might be explained by processes characteristic of the foster parents, the foster child, or perhaps both.

From the perspective of processes characteristic of foster parents, it might be that foster parents simply do not express their parental stress in ways that affect their foster children. Although foster parents in our study experienced more stress compared to parents from the general population (Goemans et al. 2016), foster parents might have the strength and ability to handle these levels of stress because they deliberately chose to be a foster parent, most likely for reasons which are highly intrinsic and altruistic (Rodger et al. 2006), and because of the training they received prior to the placement (Dorsey et al. 2008). The fact that foster parents seemed not to express their parental stress in ways that affected their foster children might reflect professionalism in foster parents, allowing them - even when they experience stress - to exhibit levels of self-control or detachment that shelter children from the negative consequences of parental stress. Furthermore, this study mainly involved stable foster placements, wherein children had resided for at least 2 years. It could be that this placement stability reflects stress levels that can be controlled by foster parents, and that are below levels connected to placement breakdown. Whereas without proper intervention, the stress experienced by biological parents can develop into unbearable levels of stress that have adverse effects on children’s development (Baker et al. 2003; Crnic et al. 2005), foster parents do not need to let this happen. They have the alternative of opting out or ending the foster placement as a final escape from their stress (Fisher and Stoolmiller 2008).

From the perspective of foster children, it can be argued that they are not susceptible to foster parent stress. Many children in foster care have previously experienced childhood adversities and have histories of neglect and abuse (Greeson et al. 2011; Oswald et al. 2010). As a consequence, it might be that foster children are - in contrast to children from the general population - not affected by relatively minor sources of distress, such as the stress expressed by their foster parents, provided the stress does not lead to parent actions that cause distress in the child. A related explanation for our finding might be emotional numbing or emotional detachment by foster children. It has been suggested that children use emotional numbing as a coping strategy in reaction to daily stressors as a reaction to previously experienced adversities (Weems et al. 2003; Wilson et al. 2011).

The overall findings of this study, supporting the presence of child-to-parent effects and the absence of parent-to-child effects, are fairly stable across different subgroups of foster children. However, there are a few differences, such as the finding that for younger foster children (<10 years) there were unidirectional prospective relations from internalizing behaviors to parental stress from both Wave I to Wave II and from Wave II to Wave III. For the entire group under study, these relations were only present from Wave II to Wave III. It seems that the child-to-parent effects for children’s internalizing behaviors on foster parent stress are generally stronger for younger children. It might be that internalizing behaviors in younger children quickly cause additional stress for foster parents because these behaviors indicate the vulnerability and complex service needs of their young foster child (Vig et al. 2005). These behaviors cannot be ignored or simply perceived as age appropriate, as might occur with older foster children.

Multigroup analyses also showed differences between foster children with different placement histories. Externalizing behaviors of foster children with one or more previous placements showed stronger child-to-parent effects than internalizing behaviors. This may be explained by the relatively strong stress inducing effects of externalizing problems on foster parents, which are related to placement breakdown (Oosterman et al. 2007). Another interesting result for foster children with different placement histories is that the bidirectional model (the model with both child-to-parent and parent-to-child effects) fit better for children with one or more previous placements than the unidirectional model with only the child-to-parent effects. It was found that for children with one or more previous placements, there was a significant unidirectional relation from foster parent stress at Wave II to foster children’s externalizing behaviors at Wave III. This seems in line with a previous study that showed that behavior problems can be both a cause and consequence of placement disruption (Newton et al. 2000): foster children who had experienced placement breakdown more often showed behavior problems (Newton et al. 2000). The current study showed that foster children’s externalizing behaviors results in increased stress in their foster parents, which might in turn place them at risk for another prematurely ending foster placement. This finding confirms previous cautions to professionals to be alert to foster parent stress and the risk of breakdown in foster children with a history of previous placements.

Despite the general lack of support for the bidirectional model, this study did show concurrent relations between foster parent stress and foster children’s behavioral problems. Furthermore, both parental stress levels and foster children’s behavioral problems were moderately stable over time. This suggests that on average, parental stress and children’s behavioral functioning do not necessarily improve over time in foster families (see also Goemans et al. 2015). The good news is that this means that behavior problems and parental stress did not become more problematic. However, given that the mean level of behavior problems fell within the borderline range, caution is warranted.

### Limitations and Implications for Future Research

Information for this study is derived from the reports of foster parents. As a consequence, same method variance might result in an overestimation of the associations between the variables of interest (Keijsers et al. 2012; O’Connor 2002). Future research should therefore include foster children’s perspectives (Johnson et al. 1995) and other informants such as teachers and professionals, to examine whether the results of the current study can be replicated. A second limitation is that the scores on behavioral functioning and parental stress did not appear to change over time, which may cause difficulties in identifying a longitudinal effect. Additional measures are recommended, especially those using other methods (e.g., interviews or observations) and more specific measures for capturing the dynamic nature of potential transactional relations (Maccoby 1992; Rothbaum and Weisz 1994), such as parent-child observations and state space grids (Granic and Hollenstein 2003; Hollenstein et al. 2004). This requires longer longitudinal studies with more waves, such as performed by Neece et al. (2012).

A third limitation is the considerable amount of attrition. Despite the use of reminders and incentives, we could not prevent dropout of more than half of our original sample. Although we compared our final sample with the group that fell out after Wave I on several important characteristics and only found one significant difference, we cannot exclude the possibility that there are important differences between those who retained and those who fell out on variables that were not measured. Attrition is a common problem within longitudinal research, and longitudinal studies on foster care are not an exception (Jackson et al. 2012). Strategies for longitudinal research with foster children as described by Jackson et al. (2012) are helpful to prevent attrition in longitudinal designs. Furthermore, researchers should be transparent in reporting their missing data, and should apply modern methods to handle missing data (Graham 2009), such as multiple imputation or FIML estimation.

To conclude, this study showed the absence of reciprocal relations between foster parent stress and foster children’s internalizing and externalizing behaviors. Foster children in general seem to influence their foster parents, but not vice versa. This can be considered as something positive: Although increased behavioral problems do heighten foster parent stress, this does not seem to continue in a downward spiral: elevated levels of parental stress do not further increase children’s internalizing and externalizing behaviors. In other words, foster parent stress does not influence their foster children’s behavioral functioning. Another positive finding is that approximately 40–45% of the foster children did not have problematic levels of behavioral problems. However, the lack of transactional relations between foster children and foster parents is surprising given the presence of transactional relations in biological parent-child dyads. Foster parents seem not to influence their foster children when it comes to regulating problem behavior. Therefore, the question is whether foster parents can, in more general terms, help their foster children benefit from their improved home environment. Given the finding that many foster children still experience behavioral difficulties, future longitudinal studies are needed to examine whether and how to boost foster children’s behavioral development.
